# Grading reflective essays: the construct validity and reliability of a newly developed Tool- GRE-9

**DOI:** 10.1186/s12909-023-04845-6

**Published:** 2023-11-16

**Authors:** Nisrine N. Makarem, Diana V. Rahme, Dayana Brome, Bassem R Saab

**Affiliations:** 1 Department of Family Medicine, American University of Beirut Medical Center, Beirut, Lebanon; 2 Psychology, American University of Beirut Medical Center, Beirut, Lebanon

**Keywords:** Reflective essays, Reflection, Medical Education, Scale development

## Abstract

**Background:**

The main objective of this study is to assess the construct validity and reliability of the Grading Reflective Essays-9 (GRE-9).

**Methods:**

This study took place in a major tertiary academic medical center in Beirut, Lebanon. 104 reflective essays written by years 1–3 residents in the department of Family Medicine at the American University of Beirut Medical Center (AUBMC) were graded by 2 trained raters who independently scored the essays using GRE-9. GRE-9 scores were then correlated with scores on communication skills OSCE stations and in-training examinations to investigate, respectively, convergent and divergent validity. One of the 2 raters scored the essays twice one month apart to assess the reliability of the GRE-9 using intra rater reliability and internal consistency.

**Results:**

There was a weak, non-significant correlation between GRE-9 score and In training examination (ITE) score (*r* = − .213, *p* = .395). There was a moderate, non-significant correlation between GRE-9 scores and the Objective structured clinical examination **(**OSCE) communication station scores (*r* = − .412 *p* = .162). The correlation coefficient between trails 1 and 2 was significant (*r* = .832, *p* = .000). Intra class correlation coefficient (ICC) analysis demonstrated almost perfect intra-rater agreement (0.819; 95% CI: 0.741–0.875) of the test ratings over time.

**Conclusions:**

GRE-9, is a short, concise, easy-to-use reliable grading tool for reflective essays that has demonstrated moderate to substantial intra-rater reliability and evidence of divergent validity. The study found non-significant correlations between reflective writing scores OSCE communication scores demonstrating a lack of relationship between reflective writing and this measure of performance.

**Supplementary Information:**

The online version contains supplementary material available at 10.1186/s12909-023-04845-6.

## Background

Reflective writing is a well-accepted tool within medical education that supports the growth of reflective capacity among medical students [1].With its consideration as an essential aspect of lifelong self-directed learning, reflective writing has become a crucial element integrated into a competence-based curriculum of the medical program [2]. The idea of reflective practice was primarily established by Schon in 1987, and it was characterized by three stages: awareness of thoughts and feelings, critical analysis of a condition, and development of a new viewpoint of the situation [3]. Reflection is also conceptualized as a process for change [4] and it is considered a fundamental aspect of enhanced learning [5] as it provides the opportunity for ‘reflection-on-action’ [6] and the demonstration of critical reflection by individuals. It follows that reflection allows the development and integration of new knowledge into practice leading to the core experience of greater professional competence [7] as it leads to improvements in empathy, communication, collaboration and professionalism [1]. A growing body of research has also highlighted the relationship between reflective capacity and the enhancement of physician competence [8, 9].

Realizing the beneficial consequences of reflection [10], medical educators have sought to explore a variety of methods for fostering and assessing reflection in learners, ranging from one-to-one mentoring [11] to guided discussions [12], digital approaches like video cases [13] and written methods like reflective portfolios, journal and essay writings [11, 14]. Reflective writing was reported to be one of the most extensively and widely used forms of reflective teaching in medical education [15, 16]. Reflective capacity within these reflective writing exercises can be assessed through various qualitative and quantitative tools [17]. Despite the presence of diverse methods, there is still a lack of best practices [17].With the proliferation of reflective writing in promoting and assessing reflection [18], the need for a valid, reliable evaluative tool that can be effectively applied to assess students’ levels of reflection was strongly called for [19].

Given that reflection is hard to measure and assess directly [14], it becomes imperative to develop simpler tools that are short, concise, include well-defined descriptors, and are easily accessible for analysis and interpretation with high level of objectivity. Since students’ approaches to learning might be affected by the type of assessment strategy used [20, 21], unreliable and invalid assessment strategies can lead to unfair results. Hence, designing a reliable and a valid assessment tool is needed.

Consequently, to serve in filling this research gap and in an effort to improve the reflective essays grading process at the American University of Beirut Medical Center (AUBMC), a new scale called the Grading Reflective Essays- 9 (GRE-9) was developed by faculty members at the Department of Family Medicine. The developed GRE-9 was found to be a reliable, concise and simple grading tool that has demonstrated moderate to substantial inter-rater reliability enabling raters to objectively grade reflective essays and provide informed feedback to residents and students [22].

Since the items of the GRE-9 scale were conceptually and thematically based on solid theoretical underpinnings and match with the four reflective levels of the REFLECT tool [19] as well as with the three essential aspects of personal reflection in the context of medical practice and education of the GRAS [2], the content validity of the scale was assumed and appeared to be satisfactory as it was grounded in reflection literature. Although GRE-9 was found to be reliable and demonstrated content validity, the construct validity of the instrument was not determined due to the small sample size. As such, investigating the validity of an instrument is of vital importance given that clear robust validity is crucial for an effective instrument [23]. Construct validity is a significant objective of validity as it mainly focuses on whether the obtained score of the instrument provides a useful and effective purpose when used in research practice [24]. A common method used in examining the construct validity of an instrument is investigating its relation to other variables. In the case of GRE-9, we tested its correlation with written examination scores and communication skills scores [25]; exploring the divergent and convergent validity of GRE-9. Research has shown that the development of reflective capacity incorporates intrinsic skills such as communication, clinical reasoning and professionalism [8, 26, 27]; thus, it is contended that scores in reflective writing will correlate with residents’ scores in measures of intrinsic skills specifically communication skills as generated by stations of objective structured clinical examination **(**OSCE) that particularly assess communication skills, but not with their scores in knowledge based examinations. Therefore, evidence of a significant correlation between OSCE score in stations that assess communication skills and reflective writing scores would be taken as evidence of convergent validity, while lack of a significant correlation with multiple-choice question on in-training examination scores that assess medical knowledge would be taken as evidence of divergent validity [28]. In an effort to additionally investigate the reliability of the GRE-9, intra rater reliability and internal consistency were also explored.

## Methodology

### Overview of Study Design

#### Sample and procedures

As part of a routine formative assessment activity, Family Medicine residents in years 1–3 training in a four-year program at the AUBMC are asked to write 1–2 reflective essays per year based on incidences from their medical practice demonstrating their ability to reflect on their learning experience. The reflective essays are not prompted and residents are asked to reflect on any incident that touched them during their practice. This provides a broader scope for reflection and bypasses the restriction on their ability to reflect when given prompts [29]. Over the academic years 2016–2020, a total number of 60 family medicine residents at AUBMC in their first to third year of residency participated in reflective writings yielding 104 reflective essays. This sample size was sufficient given that a minimum number of 40 assessment observations were required to test Cronbach’s alpha when different from 0.50 at a significance level of p < .05 and power of 0.80 [30].The sample size calculation for intra-rater reliability is computed based on the criterion value of 0.8 and the obtainment of 80% power at 5% significance level by using Power Analysis and formula for minimum sample size (n) and yielding the requirement of 70 ratings per rater [31], thus rendering the study’s sample sufficient.

Family Medicine residents sit for an annual in-training exam (ITE) conducted by the American Board of Family Medicine. This exam is in the form of multiple choice questions and aims to test the residents’ comprehensive biomedical knowledge. Residents also sit for a yearly OSCE exam that consists of 13 stations one of which assesses their communication skills.

The three types of data (OSCE score, ITE score, and GRE9 score) for each resident in years 1 to 3 across academic years 2016 to 2020 were matched to give a complete dataset, and then anonymized by using participant codes. This process was carried out by an honest broker at the department of Family Medicine. Each reflective essay was graded by two trained raters versed in the field of medical teaching, curriculum development, as well as reflective writing assessment using the GRE-9 rubric, who independently scored the reflective essays. Before starting the grading process, training sessions on the GRE-9 were conducted which included a review of the elements of the GRE-9 followed by a group discussion on how the tool should be applied. The two raters then conducted three meetings to discuss their grading of 10 randomly selected reflections, which were excluded from the study, as a way of increasing consistency across raters’ scores.

Following the training, the two raters assessed the 104 essays. To determine the final ‘reflection’ score for a given essay, the average score across the two raters was used. The average score for each participant across his/her writing samples throughout the years assessed was calculated as a final score. One of the 2 raters also rated the reflective essays another time one month after the first rating.

#### Ethical considerations

Before starting the research project, approval was sought from the Institutional Review Board (IRB) at the AUBMC. An email was sent to all residents involved informing them about the study and asking for their consent to include their reflective essays. Lack of reply to the email was considered as consent to include their anonymized reflective essays in the study. Only the reflective writings of the residents with complete ITE and OSCE data and who have consented for their anonymous data to be used in the validation analysis for the GRE-9 were utilized. Participation did not impact residents’ evaluations, which were completed before the analysis began.

#### Research Design

This study aimed to assess the different sources of evidence that support the construct validity of the study instrument. The sources of validity evidence for GRE-9 were based on investigating the relation of GRE-9 scores to other variables by testing convergent and divergent validity. Reliability of the scale was also investigated through internal-consistency and intra-rater reliability.

### Instruments

#### Grading reflective essays − 9 (GRE-9)

The GRE-9 obtained a moderate to substantial inter-rater reliability based on the Intra class correlation coefficient (ICC) krippendorff’s alpha (ICC of 0.78). The standardization of the scoring for the GRE-9 includes the following: the first 2 items of the scale, which are descriptive, are given a maximum grade of 1 whereas the rest, which are analytical, are given a maximum grade of 2. The maximum score is 16. The items are followed by a guide that clarifies each point with the aim of facilitating and standardizing the grading process. The GRE-9 consists of 9 items (Appendix).

#### In-training examination (ITE)

During their first to third residency years at the department of family medicine, residents complete an annual ITE from the American Board of Family Medicine. This consists of 200 multiple-choice questions assessing their medical knowledge. The purpose of the ITE is to provide an assessment of the residents’ progress in acquiring the medical knowledge needed to become a family physician. The ITE is scored using statistical analyses whereby there is no passing score, since the purpose of this examination is to assess the resident’s progress over the years of their residency training. Performance reports provide identified areas that the resident needs to improve and can be used to develop an individual educational plan in coordination with the residency program. Each resident is given a scaled scored that is compared to the national mean score. Because the ITE scores are contended to reflect knowledge-based performance, a low correlation is expected to emerge between the GRE-9 score and ITE score; thus, confirming evidence of divergent validity.

#### Objective structured clinical examination (OSCE)

Family Medicine residents complete an annual OSCE examination consisting of 13 stations of which one station assesses their communication skills. A clinical faculty member scores each resident on each OSCE station and evaluates his/her performance using a station-specific checklist that assesses dimensions of performance specific to that station as well as factors such as organization of the encounter and accord with the patient. A final score is given per station per resident. The total (weighted) score calculated for the single station that specifically evaluates performance on a communication challenge was extracted for each resident throughout years 2016 to 2020. A significant correlation is expected to emerge between scores on communication skills OSCE stations and reflective writing scores; thus, confirming evidence of convergent validity.

### Data Analysis

Data was analyzed using the Statistical Package for the Social Sciences (SPSS 22.0). As a definitive measure of criterion-related validity, convergent and divergent validity were investigated by using Pearson correlations coefficients (moderate = 0.3–0.7; strong 0.7–1.0). In order to determine the intra-rater reliability of the ratings, the correlation coefficients between the two grading of the same rater (R1) for the same reflective essays were also computed by using Pearson Correlation Analysis. The intra-rater reliability was also assessed using the ICC with a 95% confident intervals based on a mean-rating (*k* = 2), absolute-agreement, 2-way mixed-effects model. GRE-9 was also examined for its internal consistency using Cronbach’s alpha (α). The Spearman–Brown prophecy formula was used to determine the number of raters necessary to achieve inter-sample reliability of at least 0.90. For all inferential analyses, a p-value of ≤ 0.05 established statistical significance.

## Results

### Divergent and convergent validity

The association between the students’ reflective scores and each of the ITE and OSCE scores was investigated to assess the GRE-9 criterion-related validity. Results yielded a weak, non-significant correlation between GRE-9 score and ITE score (*r* = − .213, *p* = .395). The absence of a significant association between the two variables confirmed evidence of divergent validity. When assessing the reflective GRE-9 scores for convergent validity, results demonstrated the emergence of a moderate, non-significant correlation between GRE-9 scores and OSCE communication station scores (*r* = − .412 *p* = .162. This indicated that GRE-9’s convergent validity was not supported.

### Intra-rater reliability and internal consistency

#### Intra-rater reliability

Intra-rater reliability was determined for GRE-9 by examining the consistency of rater 1 reflection assessment at time 1 (first assessment) and at time 2 (second assessment in a one month interval). The correlation coefficient between trails 1 and 2 was significant (*r* = .832, *p* = .000). Given that the correlation coefficient was above 0.70 which refers to a sufficiently high correlation and relatively high consistency [32]; thus, indicated a strong intra-rater reliability. In order to determine the number of raters needed to achieve an almost perfect agreement (0.90-1) across the two raters, the Spearman–Brown prophecy formula was calculated and results indicated that 2 raters are enough to score 104 reflective writing samples to achieve an inter-rater reliability of at least 0.90.

Intra-rater reliability for GRE-9 was also examined using Intraclass correlation coefficients measures of agreement. Given that ICC values between 0.81 and 1.00 represent almost perfect agreement and thus high reliability according to Landis and Koch [33],

ICC analysis demonstrated almost perfect intra-rater agreement (0.819; 95% CI: 0.741–0.875) of the test ratings over time.

#### Internal consistency

Internal consistency for GRE-9 scale was assessed through an overall Cronbach’s alpha calculated for the first and second rater assessments. Given that Cronbach’s alpha of 0.70 was considered as an adequate consistency, 0.80 was considered good, and > 0.9 was considered highly consistent [34], thus, producing a low to moderate reliability (α = 0.518). Given that the length of the scale influences the value of alpha which gets reduced for short length scales, a Cronbach alpha between 0.5 and 0.7 is regarded as acceptable for such scales [35]. Table 1 presents the pattern of correlations across all measures.

The correlation coefficients computed, by using Pearson Product Moments Correlation, are presented below in Table 1.

**Table 1:** Pearson Correlations among study variables

	GRE-9 Scores
OSCE Communication Station ITE GRE-9 Time point x 2	− 0.412 − 0.213 0.832**

**. Correlation is significant at the 0.01 level (2 tailed)

## Discussion

This study demonstrates different sources of evidence to support the construct validity as well as the reliability of the GRE-9. This study is a follow-up to a prior study that was carried out by the same authors to examine the psychometrics of the GRE-9. As yielded in the primary study, content-related evidence was supported by the theory-informed construction of the study instrument since the GRE-9 rubric was based on a comprehensive analysis of relevant theoretical models of reflection as well as existing reflection assessment measures [36]. In addition to content validity, GRE-9 was found to be a reliable, concise and simple grading tool that has demonstrated moderate to substantial inter-rater reliability [22], yet the investigation of the construct validity was not determined due to the small sample size. As such, given that clear robust validity of an instrument is crucial [23], the present study aimed to further investigate the psychometrics of the GRE-9 by examining its construct validity, intra-rater reliability and internal consistency.

In accordance to examining the construct validity of the GRE-9, divergent and convergent validity were explored. Results yielded a weak, non-significant correlation between GRE-9 reflective score and ITE score; thus, confirming the evidence of divergent validity. When assessing for convergent validity, results demonstrated the emergence of a moderate, non-significant correlation between GRE-9 reflective scores and OSCE communication station scores; indicating that GRE-9’s convergent validity was not supported. Other studies in the literature that have also investigated the divergent and convergent validity of reflective tools through investigating the relationship between reflective writing scores and other measured of performance [1, 28, 37] have also yielded differential results related to construct validity. For instance, in a study aiming to investigate issues of reliability and validity in the quantitative assessment of reflective writing using an already establish reflective tool [REFLECT], results yielded a weak non-significant correlation between students’ REFLECT scores (averaged across four samples and four raters) and Year 2 MCQ examination scores which confirmed the divergent validity [1]. Study findings also yielded a weak non-significant correlation between REFLECT scores and OSCE measures; as such, failing to support the convergent validity of the scale [1]. Another similar study evaluating a newly developed scale [28] showed evidence of convergent validity for their scale. Specifically, correlations between scores in reflective portfolios and scores in both communication skills and PBL tutorials supported the evidence of convergent validity. Although, a small effect size correlation of the reflective scores in relation to written MCQ examination was obtained [28], divergent validity was not established. In another study that aimed to investigate the validity of the Reflective Practice Questionnaire (RPQ) in the Korean context to identify the level of reflection of medical students in clinical practice, the criterion validity test supported the convergent validity by yielding a positive correlation between most of the sub-factors of the Korean version of the RPQ (K-RPQ) with the Korean Self-reflection and Insight Scale (K-SRIS), which measures the attitude of daily insight, and “the Reflection-in-Learning Scale (RinLS),” which measures students’ reflective learning experiences in medical school and with “The Self-efficacy in Clinical Performance Scale (SECP)” which measures clinical performance self-efficacy [37]; in this study, divergent validity was not investigated.

The differential results related to convergent and discriminant validity in the aforementioned studies can be attributed to various factors such as content of the study instrument, levels of training of the raters, number of raters, as well as the sample size used [37]. In the present study, the emergence of a moderate, non-significant correlation between GRE-9 reflective scores and OSCE communication station scores and the disconfirmation of GRE-9’s convergent validity indicates that one or both of the variables failed to capture the intended construct well. In fact, while most theories of reflection encourage imaginative exploration of cognitive, affective, physical, and verbal experiences when making sense of ambiguous and uncertain situations, the development of a tool that breaks down reflection into discrete components restricts learners’ ability to be creative and encourages their propensity to tailor their writing to the objective of “scoring well” [38]. As such, further refinement of the reflection construct measured by GRE-9 is required in future studies. Furthermore, despite the statistical adequacy of the sample size, the non-significant correlation might also reflect the need of additional statistical power to detect a significant correlation among the variables [39].

In an effort to additionally investigate the reliability of the GRE-9, intra rater reliability and internal consistency were also explored. Results yielded strong intra-rater reliability for GRE-9 (*r* = .832), indicating that a score above 0.70 refers to a considerably high and meaningful correlation [32] and relatively a high consistency [40]. A high intra-rater agreement was also recorded (0.819; 95% CI: 0.741–0.875); which further indicates that the rater assigned similar scores to the essays in both assessments when using the GRE-9 tool. When investigating the internal consistency for GRE-9 scale, results produced a low to moderate reliability (α = 0.518). This level of reliability can be attributed to several factors. Primarily, the length of the scale is reported to influence the value of alpha which gets reduced for short length scales; thus, yielding a Cronbach alpha between 0.5 and 0.7 as acceptable for such scales [35]. Numerous studies in the literature investigated the internal consistency of reflective tools used in a medical setting; results yielded diverse results from low to high internal consistencies. For instance, the reliability of 10 sub factors Reflective Practice Questionnaire (RPQ) in the Korean context was found to be satisfactory, ranging from 0.666 to 0.919 [37]. The Groningen Reflection Ability Scale (GRAS) was developed to measure the personal reflection ability of medical students; results yielded moderate to high Cronbach’s alphas of 0.83 and 0.74 for the scale [2]. In another study investigating the internal consistency of the REFLECT scale, similar results to the present study were obtained whereby the scale items yielded poor reliability across all criteria of the tool (0.529–0.621) [38]. The low reliability in terms of Cronbach’s alpha can be attributed to the notion that the good reliability statistics are not just observed to be the result of the function of the tool solely, but also as a result of the intersection between the assessors’ application of the tool and their comprehension of what it is designed to measure [38]. This type of consensus understanding building may have taken place as a result of the inclusion of the same raters throughout testing iterations or as a result of the research team providing raters with a set of progressively-refined instructions. Although efforts were made for the raters to be prepared for the rating procedure by allowing them to go through a pre-study rater training process, yet there is a proposed possibility that this could not have been enough to address any fundamental disparities between how the raters understood the tool’s constructions [38]. Our findings have implications for the number of raters needed to obtain inter-rater reliability of at least 0.90: our study concluded the need for two raters; this is similar to Wald and colleagues [19] who proposed the use of two or three raters based on their results.

It is worth noting that the very effort of quantifying reflective writing is in itself a challenge. Charon and Hermann have argued that this effort in itself can undermine the educational value of reflective writing. They suggest that the utility of reflective writing as a channel for learning is challenged once it undergoes formative assessment. They explain that reflective writing should be used to “attain the state of reflection” and rating or grading this process can be counterproductive. When reflective writings are graded, students and residents will write with the aim of performing well rather than to simply reflect, distorting the work of reflection itself [41].

### Limitations

The limitations of this proposed study are worth pointing to. Primarily, the design of the study was restricted to years 1–3 of residency in the family medicine department at AUBMC. In this case, participants cannot be assumed to be representative of a larger population outside the study context; thus, restricting the generalizability and the replication of the study findings in different years of study and in different educational settings. Also, given that residents from first to third year of residency participated in the reflective writings, there is a possibility that those in the third-year training were more exposed to experience as well as reflective thinking processes within the field; hence, allowing for the possibility of response bias to take place.

## Conclusion

Despite the popular use of reflective essays as a tool to measure reflection, little quantitative evidence exists to support the psychometric properties of the available tools. In this study, we aimed to assess the psychometric properties of the GRE-9 as a step towards filling this research gap. Although the results did not confirm the convergent validity of GRE-9 and the scale had low internal consistency, results supported GRE-9 reliability and validity through divergent validity and high intra-rater agreement. Yet, prior to applying these findings to the evaluation of students in other medical schools, more research is required to confirm these findings and to assess additional measuring characteristics of the GRE-9. Factors, such as, content of the study instrument, levels of training of the raters, the number of raters, as well as the sample size used [28], could all be impacting the results related to the reliability and validity of the scale. An important notion is that reliability characteristics are contended to be relevant to the context in which a measurement tool is developed; as such, it becomes of paramount importance to replicate psychometric examination of such tools before applying them and using them in new educational medical context [38]. Therefore, it is important to further apply GRE-9 in different resident groups with a wider range of demographics in order to make sure that results are generalizable and to further clarify the meaning of reflection and the constructs related to this concept that is to be captured by GRE-9. Our study comes to support the notion that if medical educators are to use assessment tools to grade reflective writing, then future research should focus on the development of more reliable and valid instruments. Finally, when developing tools used to extract quantifiable data from conceptual frameworks once thought to be assessed only though qualitative methods, it is expected to be faced by conflicting results related to the scale’s reliability and validity.

### Electronic supplementary material

Below is the link to the electronic supplementary material.

Supplementary Material 1

## Data Availability

The data is available upon reasonable request by contacting the corresponding author Dr. Diana Rahme, email ds07@aub.edu.lb.
