# Supplementary material for: Grading reflective essays: the construct validity and reliability of a newly developed Tool- GRE-9
Source: BMC Med Educ. 2023 Nov 16;23:870. doi: 10.1186/s12909-023-04845-6 (PMC10655370; doi:10.1186/s12909-023-04845-6)
Supplement: Supplementary file 1 — Supplementary Material 1 [file 12909_2023_4845_MOESM1_ESM.docx]

Appendix

Scoring of GRE-9 per item and guidance for grading

| **Item** | **Not attempted** | **Partial** | **Full** |
| --- | --- | --- | --- |
| **1. What happened?**  *Guidance: State the main features of the event: persons involved, timing, place, setting, how all persons concerned acted/behaved* |  |  | 1 |
| **2. What is special about this event?**  *Guidance: State clearly the reason for choosing this event in particular. In-depth description of the dilemma, conflict, challenge posed by the event* |  |  | 1 |
| **3. Feelings when it happened**  *Guidance: Describe personal thoughts and feelings while the event was happening, emotional insight and empathy* |  |  | 2 |
| **4. What was the outcome for the concerned?**  *Guidance: Concerned include patient, significant others, health professionals, health system, society. Empathetic reflection* |  |  | 2 |
| **5. Understanding of the event**  *Guidance: Express what was good and bad about the experience; interpretation of the situation at present with justifications (factors/ knowledge influencing judgment)* |  |  | 2 |
| **6. Congruence of actions and beliefs**  *Guidance: Does the resident think he acted as per his beliefs? Was there anything that held him back from applying his beliefs? Reflection-on-action* |  |  | 2 |
| **7. New thoughts and feelings after reflection**  *Guidance: Describe resident’s new thoughts and feelings after reflecting on the case. Making meaning and analysis of the thoughts and feelings* |  |  | 2 |
| **8. Reference to old experience and others**  *Guidance: Compare to other situations, experience, others involved, and known events and facts, preferable to have reference* |  |  | 2 |
| **9. How this incident will affect future role**  *Guidance: If it arose again, how would the resident act? How did this experience change the practice for the better? Transformative learning, development of new viewpoint of the situation* |  |  | 2 |
| **Total score** |  |  | 16 |
